# Application of Latent Class Analysis to Identify Subgroups of Children with Autism Spectrum Disorders who Benefit from Social Skills Training

**DOI:** 10.1007/s10803-020-04678-y

**Published:** 2020-09-05

**Authors:** Vera Dekker, Maaike H. Nauta, Marieke E. Timmerman, Erik J. Mulder, Pieter J. Hoekstra, Annelies de Bildt

**Affiliations:** 1 Department of Psychiatry, University of Groningen, University Medical Center Groningen, Groningen, The Netherlands; 2 Accare, University Center for Child and Adolescent Psychiatry, PO Box 660, 9700 AR Groningen, The Netherlands; 3 Department of Clinical Psychology and Experimental Psychopathology, University of Groningen, Groningen, The Netherlands; 4 Department of Psychometrics and Statistics, University of Groningen, Groningen, The Netherlands; 5 Center for Intellectual Disabilities and Psychiatry, GGZ Drenthe, Assen, The Netherlands

**Keywords:** Social skills training, Autism spectrum disorder, Randomized controlled trial, Participant and intervention characteristics

## Abstract

With Latent Class Analysis applied on data of 98 children with autism spectrum disorder (ASD) (9–12 years; 17 girls) participating in social skills training (SST) in a randomized controlled trial (Dekker et al. 2019), four subgroups were detected, based on social-communicative skills before, and response patterns to training. Two subgroups improved after SST. Characterizing the subgroups based on participant and intervention characteristics showed that improvement was related to lower parent-reported perceived difficulty of social-communicative skills at start, higher verbal ability, younger age and milder symptoms of ASD and anxiety. The lowest performing non-improving subgroup participated more often in SST without parent/teacher involvement, compared to all other subgroups. Response to SST in ASD seems to vary depending on participant characteristics.

**Electronic supplementary material:**

The online version of this article (10.1007/s10803-020-04678-y) contains supplementary material, which is available to authorized users.

## Introduction

Since limitations in social communication and interaction are core characteristics of an Autism Spectrum Disorder (ASD), many of the treatment approaches for ASD focus on social communicative skills, often in the form of Social Skills Training (SST). Many studies have been conducted into the effect of SST for children and adolescents with ASD and overall, on group-level, moderate to large effect sizes have been found (Gates et al. 2017; Wolstencroft et al. 2018). Much less is known on whether specific subgroups of participants exist that benefit more from specific (characteristics of) SST. The current study aimed to contribute to this subject by investigating whether subgroups can be identified of participants who respond differently to SST with or without parent and teacher involvement, and by relating these subgroups to multiple dimensions such as participant and intervention characteristics.

The authors of two recent meta-analyses that investigated the effect of SST on group level (Gates et al. 2017; Wolstencroft et al. 2018) recommended to further investigate the benefit of specific forms of SST for specific individuals. To this end, they investigated the effect of SST in relation to several *intervention characteristics.* Further, Gates et al. 2017 also investigated *participant characteristics* in relation to effect. In their meta-analysis they could only analyze the effects on the self report measures, because of too little heterogeneity in the studies with parent report, and a too small number of studies with teacher report included.

With respect to the *intervention characteristics,* Wolstencroft et al. (2018) found that *parental involvement* appeared to have a surplus value, with large effect sizes for SST with parental involvement, and moderate ones for SST without parental involvement. Furthermore, *intensity* was related to effect size: an intensive SST, in the form of a summer camp, had a large effect size, compared to a moderate effect size for weekly sessions. Last, *duration* affected the outcome of SST in ASD, with a large effect size for SSTs that lasted over 40 h, compared to a moderate effect size of SSTs of 40 h or less. The effect of duration was corroborated in a more recent study (Jonsson et al. 2019). Gates et al. 2017 found no effect of intervention length. They found no effect of involving *peer tutors* in SST either.

Several studies investigated *participant characteristics* in relation to the effectiveness of SST in ASD in earlier studies. Consistent effects have been found for gender and cognitive ability, that is, larger effects have been found for females than males (McMahon et al. 2013; Choque Olsson et al. 2017). However, as Gates et al. 2017 indicated, many studies included too few female participants to reliably investigate the effect of gender on the outcome of SST. Further, participants with higher IQ’s benefited more from SST than participants with lower IQ’s (Herbrecht et al. 2009). It is important to note that most studies into SST in ASD were conducted with relatively high-functioning participants. Inconsistent effects have been found for age, comorbidity and medication. That is*,* older children and adolescents benefited more from SST in some studies (Mathur et al. 1998; Herbrecht et al. 2009; Choque Olsson et al. 2017), while younger children did in others (Wang et al. 2011; McMahon et al. 2013). Further, comorbid ADHD has been found to decrease the effect of SST in children with ASD (Antshel et al. 2011), or to have no effect (Deckers et al. 2016). Comorbid anxiety has been reported to increase the effect of SST in ASD (Antshel et al. 2011), to decrease the effect (Pellecchia et al. 2016), or to have no effect on outcome (Deckers et al. 2016). Finally, children on medication profited more from SST in one study (Herbrecht et al. 2009), whereas children without medication did better in another study (Frankel et al. 2007). In the meta-analysis of moderation effects of Gates et al. 2017, none of the participant characteristics were reported to significantly affect the outcome of SST in ASD when based on self report. The authors emphasized that their findings do not mean that these factors are not related to outcomes. Based on information from other raters (parent, teacher, external observer) these variables may play a role in the outcome.

It is important to note that all relations found in the studies described were based on group level effects in relation to specific characteristics. This increases our insight into factors that may affect how much children benefit from SST, yet it oversees possible specific response patterns to SST in relation to multiple characteristics. Identifying subgroups of children with ASD with similar response patterns to SST, and investigating the factors that characterize these subgroups will help us understand which specific subgroups, benefit from SST and which do not benefit or do so to a lesser extent.

To our knowledge, no studies are available that investigated whether subgroups of children with ASD can be defined that respond differently to SST. Amongst young children with ASD, research has been conducted on identifying subgroups and their development (Stevens et al. 2000; Kim et al. 2016; Paynter et al. 2018). This research has been conducted with different approaches. The first of these studies (Stevens et al. 2000) defined two subgroups of children with ASD at school-age, i.e. a higher functioning group and a lower functioning group, based on social behavior, language and cognitive functioning. As a next step, they investigated which characteristics, as measured during pre-school, were related to group-membership at school-age. They found that pre-school non-verbal cognition, language and the social domain of adaptive functioning were most related to group-membership, with the lowest functioning group at school-age having the lowest scores at pre-school. Kim et al. 2016 identified four subgroups of toddlers with ASD, a higher functioning one, a lower functioning one and two groups in between, based on their levels of social communication, rigid repetitive behaviors, nonverbal and verbal skills and adaptive functioning at one point. Their next step was to compare how these groups developed over time. The four groups differed in the stability of ASD symptoms, adaptive functioning, and verbal cognitive functioning over time. The lowest functioning group gained the least or even decreased in these areas. Paynter et al. (2018) used the development of toddlers over time to define their subgroups. They found two subgroups of toddlers with ASD based on their different patterns of response to early intervention over time: a subgroup that responded with change and a subgroup that did not change so much. The subgroups differed in cognitive, verbal and adaptive functioning, with the lowest skills in the low change group. As pointed out by Paynter et al. (2018), it is important to investigate the factors that are associated with high and low change after intervention, in order to be able to adjust intervention for those who may not respond, and to identify the factors that may help to predict who will respond to a specific intervention.

With the current study we aimed to increase the insight into patterns of response to SST of children with ASD, and their relation with multiple characteristics. Therefore, we applied a Latent Class Analysis (LCA) to a randomized controlled trial (RCT) dataset on the effectiveness of group SST with and without parental and teacher involvement, compared to each other and to care as usual (CAU) (Efficacy of Social skills Training In Autism [ESTIA]; Dekker et al. 2019). The RCT was conducted in 122 high-functioning pre-adolescents with ASD, and involved pre, post, and six months follow-up measures in all conditions (Dekker et al. 2014, 2019). The primary analyses focused on the effect of the intervention on group level. Small to moderate parent reported effects were found on some of the outcome measures (Vineland socialization and SSRS-parents; cooperation). On these measures children in both SST conditions improved more than children in the CAU from pre to post treatment (Vineland socialization SST ES = 0.39 and SST-PTI ES = 0.43; SSRS-parents SST ES = 0.43 and SST-PTI ES = 0.45). Outcome of the SST conditions did not differ from each other. On the other subscales of the parent SSRS or the specifically trained social skills, no difference between the three conditions was found. Predictors were not included in the primary analyses.

In this next step, we combined the approaches as used by Kim et al. 2016 and Paynter et al. (2018). We aimed to identify subgroups of participants based on (1) their parent reported social communicative skills before SST and (2) their patterns of response to SST over time (measured immediately and six months after SST). We did so using Latent Class Analysis, resulting in subgroups of individuals with the same patterns of response to SST. We used three measures of social communicative skills, which cover slightly different aspects, thereby allowing for detecting clinically relevant subgroups that are defined by higher-order concepts which only become visible when all characteristics are combined in one analysis. Additionally, we investigated whether and how the response patterns in the subgroups could be related to multiple participant and intervention characteristics. We examined relevant participant characteristics from the literature on SST (age, gender, verbal IQ, symptoms of ADHD, and symptoms of total and social anxiety), and from studies on change over development (verbal IQ, severity of ASD symptomatology). Assuming we would find subgroups of children with ASD with different response patterns to SST, we hypothesized that being a girl (McMahon et al. 2013; Choque Olsson et al. 2017), having a higher verbal IQ (Herbrecht et al. 2009) and lower ASD symptoms (Kim et al. 2016; Paynter et al. 2018) would be related to a more ‘successful’ response pattern. Regarding age, ADHD and social anxiety, the results have been too inconsistent to form a hypothesis. As intervention characteristic, we examined the presence or absence of involvement of parents and teachers.

## Methods

### Design

The study was based on data from an RCT on the effectiveness of group SST with and without parental and teacher involvement, with three conditions: Social Skills Training (SST; n = 47), Social Skills Training with Parent and Teacher Involvement (SST-PTI; n = 51) and CAU (n = 24) (Dekker et al. 2014, 2019). From September 2010 through September 2013, training groups had started (September and February), data collection therefore started in May 2010 (first pre test) and ended in October 2014 (last follow-up measurement).

The current study only included the children in the treatment conditions (n = 98; 47 SST and 51 SST-PTI), in order to be able to investigate their progress after SST. Three measurements were conducted: before randomization (T1), immediately after the SST (T2), and six months after the end of SST (T3). Before participation, all parents and children above 12 signed an informed consent. The study followed CONSORT guidelines for RCTs, was approved by the Institutional Review Board of the University Medical Center Groningen, and was registered in the Dutch Trial Register (NTR2405; https://www.trialregister.nl). For a detailed description of study recruitment and treatment allocation, we refer to the research protocol (Dekker et al. 2014) and the report on the findings of the primary analyses (Dekker et al. 2019).

### Participants

The participants of the ESTIA-study (n = 122) were recruited in four outpatient mental health care clinics in the northern part of the Netherlands. The current study only included the 98 participants who participated in the intervention (n = 24 were in the control condition). All were preadolescent high-functioning children with a clinical diagnosis of ASD (81 boys, 17 girls). As reported in Dekker et al. 2019 all participants met the following inclusion criteria, which were slightly adjusted from the original design registered in the trial register: (1) pre-existing clinician-based DSM-IV-TR ASD diagnosis (Autistic disorder, Asperger’s disorder, or Pervasive Developmental Disorder-Not Otherwise Specified [PDD-NOS]), based on developmental history, current problems, child observation (ADOS), and information from school in expert teams including at least a child psychologist and a child psychiatrist (original criterion: ASD diagnosis either supported by an Autism classification on the Autism Diagnostic Interview-Revised (ADI-R) or maximally two points below the cut off for Autism on the ADI-R but with an ASD classification on the ADOS); (2) the child’s clinician indicated SST as the first appropriate treatment due to social problems in school and other contexts; (3) parents and child were motivated for SST, as established during a meeting with the clinician, the parents, and the child; (4) preferably IQ ≥ 80 (original criterion: IQ ≥ 80); children with IQs slightly below 80 were included when therapists established they were able to follow an SST; (5) being in the last two and half years of primary education (original criterion: being in the last two years of primary education); (6) no physical condition affecting participation; and (7) the child could travel to the child mental health center for training. The original criteria were broadened to more closely approximate the regular decisions in clinical practice. As a result of the change in the first criterion, the clinical diagnoses in the sample had not been corroborated with classifications on standardized instruments. This indicates that the included sample functioned at or towards the less autistic end of the spectrum.

Of the 98 participants in the intervention, 65 had a DSM-IV-TR diagnosis of PDD-NOS (66%), 20 of Asperger’s disorder (20%), and 13 of autistic disorder (13%). ASD diagnoses did not differ between the two SST conditions (Pearson χ^2^ 0.25; p = 0.881). One comorbid diagnosis was established in 32.7% of the children, 3.1% had two comorbid diagnoses (20 ADHD, 20.4% of all participants; 7 a tic disorder, 7.1%; 3 an Anxiety Disorder, 3.1%; 4 an Oppositional Defiant Disorder, 4.1%; 2 other, 2%). The SST conditions did not differ in comorbid secondary diagnoses (Pearson χ^2^ 15.19; p = 0.296) or tertiary diagnoses (Pearson χ^2^ 2.94; p = 0.402). At the start of the SST the mean age was 10.9 years (SD = 0.7; range 9.5–12.7). Most children had two Dutch parents (n = 72), all had at least one Dutch parent. The characteristics of the participants at baseline are presented in Table 1.

**Table 1:** Baseline participant characteristics (N = 98, 83% male)

	Mean (SD)	Range
Age		
Years^a^	10.9 (0.7)	9.5–12.7
ADOS		
Social Affect^a^	8.3 (4.2)	0–20
Restricted and repetitive behavior^a^	1.2 (1.0)	0–5
Calibrated severity score^a^	5.5 (2.3)	1–10
ADI-R		
Social interaction^a^	14.3 (5.9)	3–27
Communication^a^	11.9 (4.7)	2–23
Restricted and repetitive behavior^a^	3.2 (2.1)	0–10
Total score^a^	31.2 (11.0)	8–55
ESTIA-TS		
Training-specific social skills^a^	70.7 (14.2)	42–106
SSRS-P		
Total^a^	35.9 (10.3)	10–59
Vineland		
Socialization^a^	81.0 (14.7)	26–118
IQ		
Verbal IQ^a^	102.9 (16.1)	72–145
Performal IQ^a^	98.0 (16.9)	60–139
Total IQ^a^	100.5 (15.7)	72–135
RCADS-C		
Social phobia^a^	7.3 (4.0)	0–16
Total anxiety score^a^	21.6 (11.5)	2–54
SNAP-IV-P		
ADHD inattention^a^	12.6 (6.6)	1–26
ADHD hyperactivity/impulsivity^a^	9.3 (5.8)	0–27

*ADHD* Attention deficit hyperactivity disorder, *ADI-R* autism diagnostic interview—revised, *ADOS* autism diagnostic observation schedule, *ESTIA-TS* efficacy of social skills training in autism—training specific, *RCADS-C* revised child anxiety and depression scale-children, *SNAP-IV-P* Swanson, Nolan and Pelham Questionnaire-parents, *SSRS-P* social skills rating scale-parents, *Vineland* Vineland adaptive behavior scales

^a^No difference between SST and SST-PTI

### Intervention

Children in both training conditions participated in a manualized SST, based on behavioral therapeutic principles and the social learning theory (Van Warners et al. 2010; internal publication), in groups of 4–6 children, led by two therapists. The training consisted of 18 sessions of 90 min. The first 15 were weekly sessions, followed by three booster sessions after two months, to maintain trained social skills. During the sessions, the children learned how they could perform social skills, based on behavioral therapeutic principles and the social learning theory. The therapists created a safe situation for the children so that they could experience that social interaction can be enjoyable. The sessions had a recurring structure: conversation, homework review, introducing a new topic, practice and role-play, new homework, and play-time. In the first four sessions the focus was on creating a safe environment for the children. In the other sessions specific topics were discussed, e.g. “asking something to someone”, “responding to bullying”.

In the SST with Parental and Teacher Involvement (SST-PTI; Van Warners and Vet 2010; internal publication) parents and teachers of the 51 children were additionally involved in the training, as opposed to SST only. Parents received 8 additional parent sessions, specifically related to the SST. Additionally, therapists met with teachers at the start of SST-PTI, followed by five telephone meetings during SST-PTI. The aim was to teach parents and teachers how to support children in practicing social skills in daily life. Therapists used instruction, behavioral exercises, and role-play. This involvement was added to advance the generalization of social skills to situations beyond the training. A more comprehensive description of the SST and SST-PTI can be found elsewhere (Dekker et al. 2014, 2019).

### Outcome Measures

We included three parent measures as indicators of social communicative skills, pertaining to the application and the perceived difficulty of social skills by their child, at three moments: before randomization (i.e. before SST; T1), immediately after SST (T2), and six months after the end of SST (T3). The measures used in this study rate social performance rather than social competence.

#### Vineland Adaptive Behavior Scales

The Vineland Adaptive Behavior Scales—Survey version (Vineland; Sparrow et al. 1984) is a semi-structured parent interview. We used the “Socialization” domain (66 items), because this domain corresponded most with the social skills in the SST, in its Dutch version (De Bildt and Kraijer 2003). Raw scores were used (range 0–132), since no Dutch normed scores are available for children with IQ’s above 70. In order to clinically interpret raw scores on the Vineland, estimated age equivalents had been developed (De Bildt and Kraijer 2003), and these will be used in the current study to interpret change. The psychometric properties of the Vineland are considered good (Sparrow et al. 1984; De Bildt and Kraijer 2003).

#### Social Skills Rating System

The Social Skills Rating System (SSRS, Gresham and Elliott 1990; Van der Oord et al. 2005) is a standardized questionnaire about general social skills in home situations. Parents rated the frequency of behavior on a 3-point Likert scale. We used the total raw score of the 38-item parent-version (SSRS-P), since no Dutch normed scores are available. The total raw score may range from 0 to 76. For interpretation of change, we applied the American norm scores for boys in Elementary schools. The psychometric properties of the SSRS are good (Gresham and Elliott 1990).

#### ESTIA-Training Specific

The ESTIA-Training Specific (ESTIA-TS; Vet et al. 2010; unpublished questionnaire) is a parent questionnaire about the difficulty of the specific social skills trained during the SST for the child. It consists of 30 training specific social skills that are taught explicitly (e.g., ‘recognizing emotions’, ‘asking something to someone’ and ‘saying no when you don’t want something to happen’). The parents reported how difficult each of the social skills was for their child, on a 4-point Likert scale (total range 30–120).

With these three instruments we aimed to measure slightly different aspects of social communicative skills. That is, we aimed to measure the specific skills that were explicitly taught during the SST, focusing on the perceived difficulty as reported by parents (ESTIA-TS). Moving further away from the training we aimed to measure the actual application of social skills in daily life, with a slight difference in level of generalization of application between the Vineland and the SSRS.

#### Participant Characteristics

Various participant characteristics were collected that have been shown to impact the effect of SST in earlier research. *Verbal cognitive ability* was assessed with the Dutch Wechsler Intelligence Scale for Children- 3th edition (WISC-III, Wechsler 1999; Dutch version, Kort et al. 2005). *Severity of ASD symptoms* was measured with the Autism Diagnostic Observation Schedule (ADOS, Lord et al. 1999; Dutch version, De Bildt and De Jonge 2008) and Autism Diagnostic Interview-Revised (ADI-R, Rutter et al. 2003; Dutch version, De Jonge and De Bildt 2007). For the ADOS, we applied the calibrated severity scores (CSS; Lord et al. 2012). Symptoms of ADHD were measured with the two subscales *Inattention* and *Hyperactivity/impulsivity* of the 26-item parent-version of the Swanson, Nolan, and Pelham questionnaire (SNAP-IV, Swanson 1997). *Symptoms of Social Phobia, and Total Anxiety* were measured with the Social Phobia subscale and the total score of the five anxiety subscales of the child-version of the Revised Child Anxiety and Depression Scale (RCADS; Van Oort et al. 2009). All data were categorized as described in Table 2.

**Table 2:** Categorization of the participant characteristics

Participant characteristic	Measurement	Categories
Gender		1. Male 2. Female
Age		1. year 2. 10 year 3. 11 year 4. 12 year
Verbal cognitive ability	WISC-III	1. Below average (< 90) 2. Average (90–110) 3. Above average (> 110)
Severity of ASD symptoms	ADOS	1. inimal evidence ASD (CSS 1–2) 2. Low level ASD (CSS 3–4) 3. Moderate level ASD (5–7) 4. High level ASD (8–10)
	ADI-R	1. No symptoms of ASD/AD 2. Symptoms of ASD as defined by Risi et al. (2006)^a^ 3. Symptoms of AD as defined in the ADI-R manual (Rutter et al. 2003)^b^
Symptoms of ADHD	SNAP-IV Inattention	1. Below population mean scorec 2. Above population mean score Male means 9 y 9.09; 10 y 7.02; 11 y 6.48 Female means 9 y 4.5; 10 y 4.68; 11 y 8.37 For children aged 12, no population data were available^d^
	SNAP-IV Hyperactivity/impulsivity	1. Below population mean scorec 2. Above population mea score Male means 9 y 7.2; 10 y 6.12; 11 y = 6.93 Female means 9 y 3.78; 10 y 3.96; 11 y 6.12 For children aged 12, no population data were available^d^
Symptoms of Social Phobia	RCADS	1. Below population mean scoree 2. Above population mean score Male and female means 10 y 7.2; 11–12 y 6.84 For children aged 9, no population data were available^f^
Symptoms of total anxiety	RCADS	1. Below population mean scoree 2. Above population mea score Male and female means 10 y 22.0; 11 y 20.33; 12 y 18.37 For children aged 9, no population data were available^f^

^a^Child meets one of the following three criteria (a) cut-off social interaction domain ≥ 10 and cut-off communication domain ≥ 6; (b) cut-off social interaction domain ≥ 8 and cut-off communication domain ≥ 8; (c) social interaction domain ≥ 9 and communication domain ≥ 7)

^b^Cut-off social interaction domain ≥ 10 and cut-off communication domain ≥ 8

^c^Categorization based on population data from American children with a Caucasian background, separated for age and sex (Bussing et al. 2008)

^d^Excluded for the analyses regarding ADHD symptomatology

^e^Categories were based on data in the Dutch population for each age (Van Oort et al. 2009)

^f^Excluded for the analyses concerning symptoms of Social Phobia and Total Anxiety

#### Intervention Characteristics

*Parent and teacher involvement* was based on the condition that participants were randomized to: SST with or without additional parent and teacher sessions.

### Analyses

We used Multilevel Latent Class Growth Analysis (MLCGA; Muthén 2004; Palardy and Vermunt 2010) to identify distinct subgroups of participants based on their level and perceived difficulty of social communicative skills as reported by parents at start of SST and their response to SST. In the MLCGA the three outcome measures (Vineland Socialization, SSRS-P, and ESTIA-TS) at the three moments (before (T1), immediately after (T2) and six months after end of SST (T3)) were jointly analyzed, using for each outcome variable a regression equation with normal error, including an intercept and two dummy variables. The latter were coded such that the intercept referred to T1, and the slopes for the dummy variables referred to the difference between T1 and T2, and to the difference between T2 and T3. MLCGA models with one to seven classes were estimated (i.e., response patterns). From these models, we selected the model with a minimal class size of 10% and the lowest value of the Bayesian Information Criterion (BIC; Lanza et al. 2007). The BIC indicates a model with an optimal balance between fit and model complexity. Of the selected model, we calculated within group effect sizes (ES) for all slopes and classes (i.e., per class change between T2-T1 and/or T3-T2, as $d= \frac{\left({\widehat{\mu }}_{Tj}-{\widehat{\mu }}_{T(j-1)}\right)}{{\widehat{\sigma }}_{T1}}$, with *j* = 2,3). ES were based on the estimated model, and were interpreted as ES = 0.2 as small, ES = 0.5 medium, ES = 0.8 large and ES = 1.3 very large (Cohen 1988), and applied as indicators for the clinical relevance of the findings.

For each participant we established the posterior probability of membership of each class based on the selected model above. Each participant was assigned to the class that matched with his or her highest probability.

In a follow-up analysis, we predicted the probability of class membership with each of the selected participant and intervention characteristics via univariable regression analyses, using the so-called three-step procedure as described in Vermunt and Magidson (2013). The participant and intervention characteristics were categorized as described in Table 2. These analyses provide insight into the distribution of the characteristics in each class, indicating whether differences between classes can be detected, based on other characteristics than outcome. For the characteristics that appeared to be significant, we performed a post-hoc analysis, to examine possible differences among the different classes with paired comparisons. Additionally, the characteristics that appeared to be significantly related to class membership were included in a multivariable regression analysis, to assess their unique contribution to predict class membership. The analyses were performed with Latent Gold 5.0 (Vermunt and Magidson 2013), using all available data.

## Results

### Missing Data

For the first measurement (T1) no missing data existed. Four participants dropped-out after the first measurement, but before the start of the actual SST, for these participants T2 and T3 data were not available. Additionally, at T2, two parents did not complete the ESTIA-TS and one did not complete the ESTIA-TS and the SSRS. At T3, for four additional participants, no data were available and two parents did not complete the ESTIA-TS and the SSRS.

### Multilevel Latent Class Growth Analysis

A four-class model was selected and interpreted, as this model had the lowest BIC (see Table 3) and a minimal class size of 10%, with the smallest of the four classes representing 17.4% of the children.

**Table 3:** BIC values for the MLCGA models with one up to seven classes

Number of classes	1	2	3	4	5	6	7
BIC	6791.2	6580.9	6531.3	**6528.4**	6531.4	6550.5	6572.1

The smallest BIC value is printed in bold face

*BIC* Bayesian information criterion

### Interpretation of the Classes

**Fig. 1:**
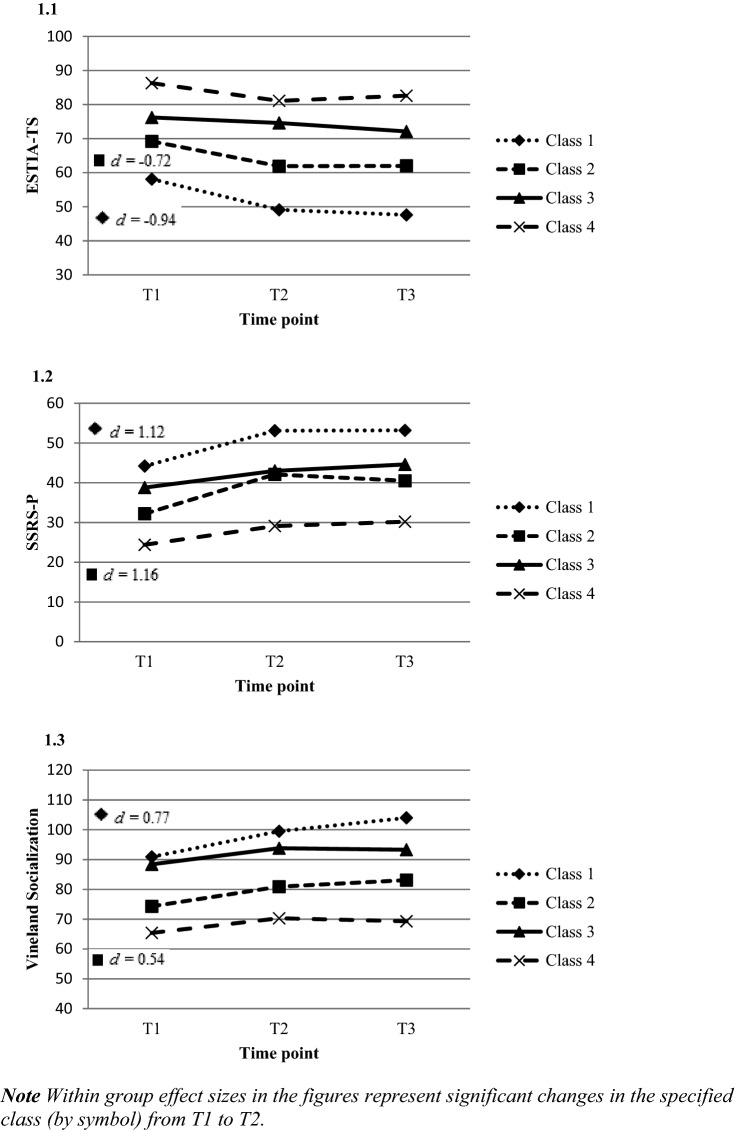
Perceived difficulty and level of social skills at start and during SST for each class

The four classes represent four underlying subgroups of children with ASD with different levels and perceived difficulty of social skills as reported by parents at start and different patterns of response during and after SST. Table 4 and Fig. 1 present the information for interpreting and naming the four classes.

**Table 4:** Perceived difficulty reported by parents and level of social skills at start and during SST for each class

Dependent variable	Class	Intercept	SE intercept	Slope T1–T2 (ES)^c^	SE Slope T1–T2	Slope T2–T3 (ES)^c^	SE Slope T2–T3
ESTIA−TS difficulty	Class 1	58.1***^a^	1.8	− 9.0*** (d = − 0.94)	2.9	− 1.5 (d = − 0.16)	2.7
	Class 2	69.2***	1.9	− 7.3** (d = − 0.72)	2.6	0.1 (d = 0.01)	2.7
	Class 3	76.2***^b^	2.1	− 1.6 (d = − 0.15)	2.9	− 2.5 (d = − 0.24)	2.9
	Class 4	86.3***^b^	2.5	− 5.2 (d = − 0.50)	3.3	1.5 (d = 0.15)	3.4
SSRS − parents total	Class 1	44.2***^a^	1.5	8.9*** (d = 1.12)	2.1	0.1 (d = 0.01)	2.2
	Class 2	32.2***	1.6	9.9*** (d = 1.16)	2.2	− 1.6 (d = − 0.19)	2.2
	Class 3	38.8***^a^	1.8	4.2 (d = 0.47)	2.3	1.6 (d = 0.18)	2.5
	Class 4	24.4***^b^	1.9	4.7 (d = 0.60)	2.7	1.1 (d = 0.14)	2.7
Vineland socialization	Class 1	90.9***^a^	2.1	8.6** (d = 0.77)	2.9	4.5 (d = 0.40)	3.0
	Class 2	74.3***^b^	2.3	6.6* (d = 0.54)	3.0	2.2 (d = 0.18)	3.1
	Class 3	88.4***^a^	2.4	5.4 (d = 0.46)	3.3	− 0.5 (d = − 0.04)	3.3
	Class 4	65.4***^b^	2.8	4.9 (d = 0.42)	3.7	− 1.0 (d = − 0.09)	3.8

^*^p < 0.05

^**^p < 0.01

^***^p < 0.001

^a^Significantly better score than the mean across all children before SST

^b^Significantly weaker score than the mean across all children before SST

^c^Within group effect size (ES)

Class 1 is characterized by significantly higher levels of social communicative skills (SSRS percentile 19 and Vineland Socialization age equivalent 6y8m – 6y10m) and significantly lower perceived difficulty of these skills as reported by parents than the mean across all children before the SST, even though the levels of these skills are still low as expressed in the norm scores or age equivalents. Additionally, the children significantly improved in their social communicative skills (SSRS: from percentile 19 to percentile 53 from T1 to T2, ES 1.12; and Vineland Socialization: improvement of 14 months between T1 and T2, to 7y10m/8y, ES 0.77) after SST and their perceived difficulty as reported by parents significantly decreased after SST (ES − 0.94). The children showed no further change six months after SST. We named this class *best performers (high starters, perceived difficulty below the mean; improvement)* and it consisted of 28.6% of the children.

Class 2 is characterized by significantly lower scores on Vineland Socialization (age equivalent 5y2m) than the mean across all children before SST. The perceived difficulty of social skills as reported by parents was similar to the mean across all children before SST. Additionally, the children significantly improved in their social communicative skills after SST (SSRS: from percentile 2 to percentile 14 between T1 and T2, ES 1.15; and Vineland Socialization: improvement of 6–8 months between T1 and T2, to 5y8m/5y10m, ES 0.53) and their perceived difficulty as reported by parents significantly decreased after SST (ES – 0.71). The children showed no further change six months after SST. We named this class *improvers* (*low starters, mean perceived difficulty; improvement),* and it was the biggest class with 29.2% of the children*.*

Class 3 is characterized by significantly higher levels than the mean across all children before SST on social communicative skills (SSRS percentile 8 at T1; and Vineland Socialization age equivalent 6y6m) and significantly higher perceived difficulty of these skills, as reported by parents, than the mean across all children before SST. No significant change appeared in skills (SSRS from percentile 8 to percentile 18 between T1 and T2; Vineland Socialization: improvement of 4 months between T1 and T2, to 7y2m) or perceived difficulty as reported by parents, immediately and six months after training. We named this class *stable class (high starters, perceived difficulty above the mean; no improvement),* and it consisted of 24.8% of the children*.*

Class 4 is characterized by significantly lower levels of social communicative skills (SSRS below percentile 2 at T1; and Vineland Socialization age equivalent 4y6m) and significantly higher perceived difficulty of these skills, as reported by parents, than the mean across all children before SST. No significant change appeared in skills (SSRS: did not exceed below percentile 2 from T1 to T2; Vineland Socialization: improvement of 4 months between T1 and T2, to 4y10m) or perceived difficulty as reported by parents, immediately and six months after training. We named this class *poor performers (low starters, perceived difficulty above the mean; no improvement)*, and it represented the smallest proportion with 17.4% of the children*.*

**Fig. 2:**
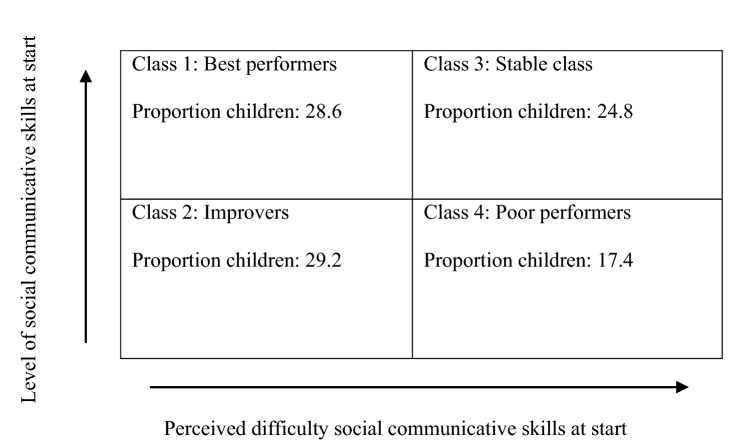
Distribution of children over the classes, related to level and perceived difficulty of social communicative skills at start

Of the four classes, the two classes with average or lower perceived difficulty of social skills as reported by parents (i.e., Classes 1 and 2) showed significant improvement of high to very high effect size during the SST. The other two classes, with higher perceived difficulty as reported by parents (i.e., Classes 3 and 4) showed no significant improvement during the SST, independent of the actual level of social communicative skills at start; yet the effect sizes of improvement during SST were small to medium, suggesting that this lack of significance may be due to a too small power. None of the four classes showed a clear change from T2-T3, as indicated by nonsignificant tests and generally very small effect sizes. See also Fig. 2.

### Relationship of Participant and Intervention Characteristics with Class Membership

**Fig. 3:**
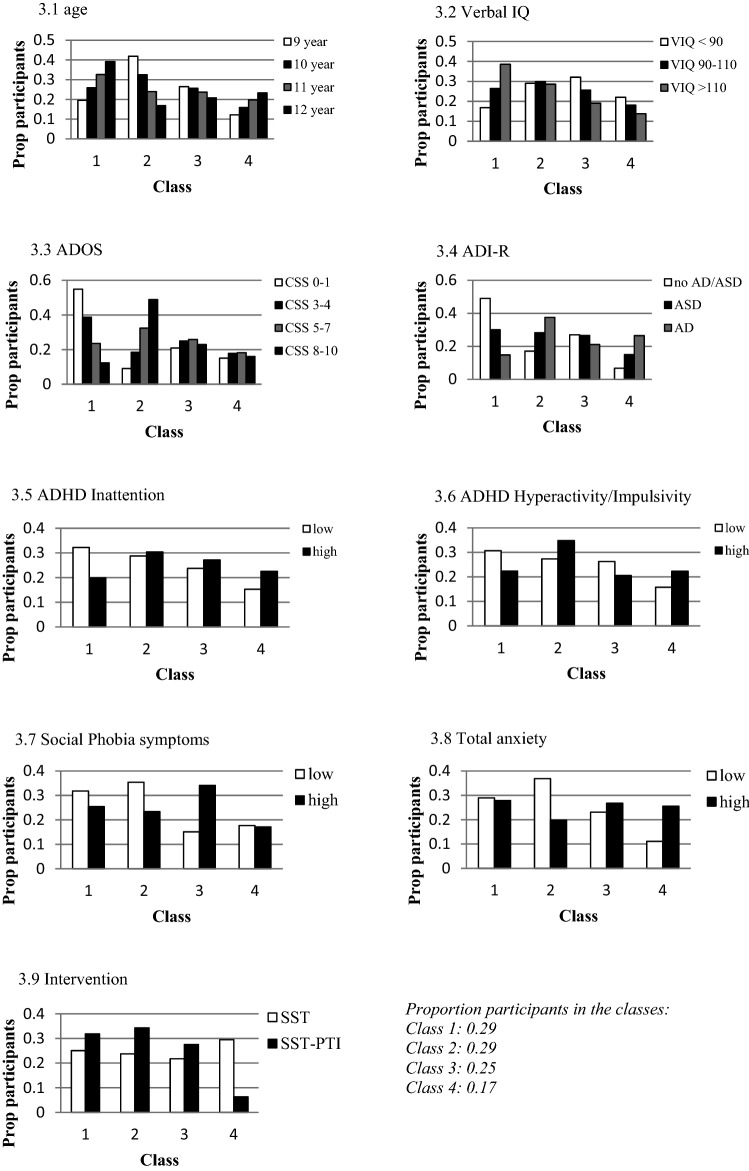
Distribution of participant and intervention characteristics over the classes

In Table 5, we present the results of the univariable regression analyses predicting class membership based on each *participant and intervention characteristic.* The mean scores on these characteristics in each of the four classes is presented in supplementary Table 1. We here report the characteristics that showed a significant relationship with class membership. See also Fig. 3.

**Table 5:** Relationship between participant and intervention characteristics and class membership

Class	1 vs 2	1 vs 3	1 vs 4	2 vs 3	2 vs 4	3 vs 4
Characteristic						
Gender	–	–	–	–	–	–
Age	**	–	–	–	*	–
Verbal IQ	–	**	**	–	–	–
ASD symptoms: ADOS	***	*	*	*	*	–
ASD symptoms: ADI-R	***	–	***	*	–	***
ADHD inattention	**	–	**	–	–	–
ADHD hyperactivity/impulsivity	*	–	**	**	–	***
Social phobia	–	**	–	**	–	*
Total anxiety	–	–	*	*	***	–
Parent and teacher involvement	–	–	***	–	***	***

^*^ p < .05; ** p < .01; *** p < *.001;*—no significant difference between the classes

For age, ‘improvers’ were the youngest and differed significantly from ‘best performers’ and ‘poor performers’. On verbal ability, ‘best performers’ scored higher than the ‘stable class’ and ‘poor performers’. On ASD symptoms measured with the ADOS, ‘best performers’ showed the least severe symptoms and differed significantly from all other three classes. ‘Improvers’ showed the most severe symptoms, compared to all other classes. On ASD symptoms measured with the ADI-R, ‘poor performers’ showed the most severe symptoms and differed significantly from ‘best performers’ and ‘stable class’. ‘ADHD Inattention and Hyperactivity/impulsivity symptoms were most apparent among the ‘improvers’ and ‘poor performers’, compared to both other classes for Hyperactivity/impulsivity, and to best performers on Inattention. Social phobia symptoms were most present in the ‘stable class’ compared to all other classes. ‘Stable class’ also showed more total anxiety symptoms than ‘improvers’, as did ‘poor performers’ compared to ‘improvers’ and ‘best performers’. Gender was the only participant characteristic that was not significantly related to class membership.

On the intervention characteristic (parent and teacher involvement), the ‘poor performers’ participated more often in SST without parent and teacher involvement than in SST with parent and teacher involvement, compared to all other classes.

In the multivariable regression we included all participant and intervention characteristics except gender. All but ADHD inattention symptoms, appeared to be significant in the multivariable regression analysis and thus relate uniquely to class membership.

## Discussion

In the current study, four subgroups of children with ASD were identified with MLCGA, based on their level and perceived difficulty of social performance before SST as reported by parents, and on their progress over time (measured immediately and six months after SST). Two of these subgroups improved after SST (6–14 months on the Vineland Socialization domain in the 6 months between pre and posttest, and 12–34 percentiles on the SSRS), the other two showed no significant improvement (4–6 months on the Vineland Socialization and 0–10 percentiles on the SSRS). None of the subgroups improved or declined between the end of SST and follow-up six months later. These findings indicate an effect of SST for specific subgroups of children with ASD. In our primary analyses only small to moderate parent reported effects were found of the intervention on group level, and only on part of the outcome measures (Vineland socialization and SSRS-parents; cooperation; Dekker et al. 2019). The current analysis indicates that two specific subgroups improved on all three outcome measures (57.8% of the children), whereas two subgroups (42.2%) did not improve.

The improvement in the two improving subgroups seems of clinical relevance and is most clear in the first, with the highest improvement (large to very large effect for ESTIA-TS and SSRS, medium (close to large) for Vineland Socialization). The other improving subgroup improved to a lesser extent (medium effect size for ESTIA-TS, large to very large for SSRS, large for Vineland Socialization). Note that all subgroups had low levels of social behavior at start, which stayed relatively low after SST, even after improvement.

The findings of improving subgroups become even more interesting when relating the response patterns in the subgroups to the multiple participant and intervention characteristics in order to detect differences between them, based on other characteristics than outcome. Improvement did not seem to be related to level of social performance at start, as the two improving subgroups differed in their level of social performance at baseline (above mean versus low to mean scores). Similarly, the non-improving subgroups differed on social performance compared to each other. In contrast, perceived difficulty of social skills for the child, as reported by parents, showed to be more crucial, as the improving groups had the lowest perceived difficulty (low and mean scores, respectively), and the non-improving subgroups showed high perceived difficulty of these skills. This may be due to the possibility that two children can perform equally well in how and how often they interact (expressed in comparable scores on actual performance as measured with SSRS/Vineland Socialization), even though they can differ enormously in the difficulty they have in reaching that behavior (expressed in significantly different scores on perceived difficulty as reported by parents on the ESTIA-TS). The latter may be of greater importance for benefiting of SST. Perhaps the children with ASD whose parents reported that their children did not perceive difficulty in interacting with others, may already interact more and benefit from the current SST by changing their manner of doing so to a more adequate one, without having to increase the frequency with, or the situations in which they interact.

Additionally, regarding participant characteristics, the current study did not find an effect of *gender* in relation to outcome on SST or basal social performance level. Earlier studies showed larger effects of SST for females than for males (McMahon et al. 2013; Choque Olsson et al. 2017). The absence of such a finding in the current study may be due to limited power to detect gender differences, as we included relatively few females (only 17%). Many earlier studies also included too few female participants to reliably investigate the effect of gender on the outcome of SST (Gates et al. 2017), so more research into the effect of SST in relation to gender should be conducted. Younger *age* was related to improvement, but only in the subgroup with lower levels of social performance at start. That is, younger children with lower levels of social skills improved more from training than older ones with lower levels of social skills. Former research did not result in firm conclusions on age and its influence on outcome in SST for ASD, and our study does not lead to a final conclusion either. We had a rather narrow age range in our study (9–12 years), which may decrease the influence of age on the outcomes of SST and the power to detect such an influence. *Verbal cognitive ability* was highest in the improving subgroup with high levels of social performance at start, illustrating the relatively high level of functioning of these children. Less verbally able children were more often in the non-improving subgroups, either with higher or lower levels of social communicative skills at start. Even in this relatively high functioning sample, this finding seems to corroborate former research concluding that participants with higher developmental levels benefit more from SST (Herbrecht et al. 2009) or from early intervention and development (Stevens et al. 2000; Kim et al. 2016; Paynter et al. 2018) than participants with lower developmental levels. This finding probably also relates to the character of the currently studied intervention that has a high verbal component in its explanation of skills and the homework assignments. However, larger samples with larger variation in are needed to further investigate the role of verbal cognitive ability.

Contrary to our hypothesis, *ASD symptom severity* was not unequivocally related to improvement. The improving subgroup with high levels of social performance at start showed the least severe ASD symptoms on the ADOS and ADI-R, also compared to the subgroup with high levels of social performance at start without improvement (for the ADOS). This is in line with findings on early intervention and development (Stevens et al. 2000; Kim et al. 2016; Paynter et al. 2018). However, we also identified a subgroup with lower social performance at start that improved, even though this subgroup showed the most severe symptoms on the ADOS within our sample and the most severe but one on the ADI-R within our sample. We would have expected the scores on the ADI-R and ADOS at start to be more directly related to the response pattern to SST, and thus to contribute more clearly to describing the subgroups that benefit more or less from SST. Perhaps the fact that the current sample only consisted of relatively high functioning children with ASD, including many with PDD-NOS (66%) and Asperger’s Disorder (20%), and that the mean scores on ADI-R and ADOS were relatively low, has led to a too small range and variation in ASD symptom severity in the current sample leading to limited power to allow drawing strong conclusions.

Regarding comorbidity, the relation found between *symptoms of ADHD* and subgroups seems to indicate that ADHD affects basal levels of social performance in the current sample, yet not outcome. Children with higher scores on inattention and hyperactivity/impulsivity symptoms had lower social performance levels at start than children with lower scores on these domains; however, improvement is not related to the level of these symptoms. This is in line with some earlier research reporting no effect of ADHD on outcome of SST (Deckers et al. 2016), yet it is in contrast with other findings (Antshel et al. 2011) reporting a decreasing effect of ADHD on the effect of SST in children with ASD. In the current sample inattention symptoms were relatively frequent in general. *Anxiety* was also related to subgroups, indicating an effect on outcome of social performance, independent from social performance levels at start. The low starting, no improvement subgroup had higher scores on total anxiety than the subgroups that did improve, including the low starting subgroup that improved. Perhaps the level of anxiety explains why some children with ASD with lower basal social performance benefit from SST (lower level of anxiety), whereas others do not (higher level of anxiety). Earlier research was inconclusive on this issue, and our study seems most in line with the study that reported that anxiety decreased the effect of SST (Pellecchia et al. 2016). Notably, the level of anxiety seemed to vary over the subgroups in the classes in a similar way as the perceived difficulty of social performance as reported by parents. The poor performers subgroup (low starters, no improvement) contained children with higher levels of anxiety and higher perceived difficulty as reported by parents, the best performers subgroup (high starters, improvement) contained children with lower scores on each. The improvers subgroup (low starters, improvement) consisted of children with mean perceived difficulty as reported by parents compared to the whole group and low levels of anxiety, and the stable subgroup (high starters, no improvement) showed higher perceived difficulty as reported by parents and higher anxiety (compared to the improvers). Possibly anxiety affects how children participate in SST. Anxious children may hesitate to perform crucial parts of training during the sessions (role play, discussing homework, answering questions, etc.) or even more so beyond the session (practice in school or at home), or doing so may take much more energy that they cannot spend on the actual content of the assignments. This could lead to less benefit from training for these children in its current form. Of note, as mentioned for all other participant characteristics, comorbidity should be addressed in larger studies in order to optimize the power to detect it’s influence.

Regarding the intervention characteristic presence or absence of *parent and teacher involvement*, we found this to be related to the subgroup with low skills at start and no improvement. That is, more children in this subgroup participated in SST without parent and teacher involvement than in SST with such involvement. In the comparison between the three conditions of the RCT that the current paper was based on, we found no difference across all participants between the effect of SSTs with or without parent and teacher involvement compared to CAU reported by parents (Dekker et al. 2019). Yet, taking into account individual differences, the current study indicates that no formal parent and teacher involvement negatively affects outcome on SST for those children with poor social communicative skills and high perceived difficulty of these skills as reported by parents at start.

In order to understand what factors affect improvement after SST, we compared the subgroups with similar basal levels of social performance. In the subgroups with relatively high basal levels, verbal cognitive ability, lower ASD symptoms on the ADOS and ADI-R, and lower social phobia symptoms seem to be related to improvement after SST. Additionally, in the subgroups with low basal levels, improvement seems to be most clearly related to lower perceived difficulty as reported by parents, younger age, lower anxiety and presence of parent and teacher involvement.

### Strengths and Limitations

The discussion of the findings in our study indicates several strengths that contribute to the existing literature; from the manualized training with and without parent and teacher involvement, to the variety of participant characteristics that we could include in the analyses. However, the current study also had several limitations.

First, for the type of analyses, the sample size of the children included is relatively small, which limits the power to detect the role of all participant and treatment characteristics.This implies that further studies using a similar design are needed to corroborate the current findings. Second, we considered a relatively high functioning sample, both with respect to ASD symptom level and to verbal cognitive level. Although the sample represents children diagnosed with ASD (based on DSM-IV-TR criteria) who were referred for SST in clinical practice, their clinical diagnosis had not been corroborated by standardized instruments, such as the ADOS or ADI-R. Additionally, the majority of children (66%) received a clinical diagnosis of PDD-NOS. This impacts the generalizability of the findings as the results of the current study may perhaps be more applicable to children in clinical practice with autism traits and social skills impairments rather than a strict ASD diagnosis as defined in the DSM-5 criteria. Third, we could only include parent reported measures, completed by parents who were only blind to condition at pretest, which may have led to bias, since they knew in which condition their child had participated after training. We did not include objective measures, completed by independent raters. Adding blinded outcome measures by someone not involved in the training (unlike parents) is currently an important focus of research, in order to increase the highly needed insight into the objective effect of SST or other interventions for children with ASD. However, none was available at the time the current study started. For this aim we had developed a measure of naturalistic behavior in school, observed by an independent rater (Dekker et al. 2016), yet questions have risen on the feasibility during the process of data collection. Other measures are for younger children (e.g. the Brief Observation of Social Communication Change; Grzadzinski et al. 2016). The current study still is informative and adds to the meta-analysis of Gates et al. 2017, as they focused on the relation between participant characteristics and self reported improvement. Additionally, we used the ESTIA-TS to measure perceived difficulty of social performance as reported by parents, a simple, yet non-validated instrument. Fourth, we could not investigate medication as a participant characteristic, or duration and intensity as intervention characteristics. We did not have data on the exact medication at baseline of the children. Additionally, all children participated in the same 15 sessions of 90 min (22.5 h of duration), in the same weekly pattern. Our duration and the intensity belong to the less effective end according to the literature (Wolstencroft et al. 2018). Last, the concept measured as the outcome in the current study is closer to social performance than social competence. Social performance is a narrower concept, yet it is in line with the aims of the training, i.e. applying social skills in role play and real life.

The current study provides insight into differences between subgroups of children with ASD in their response to SST, even within a relatively homogeneous sample, thereby specifying the effects found on group level. By doing so the current study contributes to the desired development towards personalizing SST for children with ASD. As improvement was not related to level of social communicative skills at start of the intervention but showed to be related much more to perceived difficulty of social communicative skills as reported by parents at start, the subgroups with higher perceived difficulties and anxiety may benefit more from an SST that focuses explicitly on perceived difficulty and anxiety in interaction. Additionally, subgroups with lower verbal levels probably benefit more from a training that addresses learning of social communicative skills through other means than mainly verbally. Last, explicit parent and teacher involvement in SST only seems to enhance the outcome of training for children with low levels of social communicative skills before training. Adapting training from the perspectives of specific needs of children with ASD may add to the benefit of SST. However, the current study concerned only one training (with and without parent and teacher involvement) and a specific sample. Replication in other samples and with other training approaches is needed, refining our understanding of what works for whom in SST, in order to reach the ultimate goal of personalizing social skills treatment for children with ASD.

## Electronic supplementary material

Below is the link to the electronic supplementary material.

Supplementary file1 (DOCX 20 kb)
