# Supplementary material for: Application of Latent Class Analysis to Identify Subgroups of Children with Autism Spectrum Disorders who Benefit from Social Skills Training
Source: J Autism Dev Disord. 2020 Sep 5;51(6):2004–18. doi: 10.1007/s10803-020-04678-y (PMC8124042; doi:10.1007/s10803-020-04678-y)
Supplement: Supplementary file 1 — Supplementary file1 (DOCX 20 kb) [file 10803_2020_4678_MOESM1_ESM.docx]

Supplementary table 1. Mean scores (SD) on all participant characteristics in the four classes, based on the modal class membership

|  | | **Class 1** | | **Class 2** | | **Class 3** | | **Class 4** | |
| --- | --- | --- | --- | --- | --- | --- | --- | --- | --- |
|  | | mean (SD) | Range | mean (SD) | Range | mean (SD) | Range | Mean (SD) | Range |
| **N** Male:Female | | 22:7 |  | 22:6 |  | 22:2 |  | 15:2 |  |
| **Treatment condition** SST:SST-PTI (*n*) | | 12:17 |  | 11:17 |  | 10:14 |  | 14:3 |  |
| **Age** Years | | 10.9 (0.7) | 9.6-12.4 | 10.8 (0.6) | 9.7-12 | 10.9 (0.9) | 9.5-12.7 | 11.1 (0.6) | 9.9-12.6 |
| **Vineland** | Socialization | 90.7 (9.6) | 68-108 | 74.1 (8.9) | 55-95 | 88.8 (10.7) | 71-118 | 64.9 (15.1) | 26-90 |
| **SSRS-P** | Total | 44.0 (8.1) | 25-59 | 31.9 (7.1) | 15-43 | 39.1 (7.5) | 28-53 | 24.1 (6.6) | 10-35 |
| **ESTIA-TS** | Training-specific social skills | 58.1 (9.0) | 42-74 | 69.6 (9.4) | 49-86 | 76.3 (10.6) | 53-97 | 86.0 (13.6) | 66-106 |
| **ADOS** | Social Affect | 6.7 (3.1) | 0-13 | 9.4 (4.0) | 3-19 | 8.4 (4.7) | 2-20 | 8.9 (4.7) | 2-20 |
|  | Restricted and Repetitive behavior | 1.0 (0.8) | 0-3 | 1.5 (1.2) | 0-5 | 1.3 (1.0) | 0-4 | 0.9 (0.9) | 0-3 |
|  | Calibrated Severity Score | 4.6 (2.1) | 1-8 | 6.5 (2.0) | 3-10 | 5.5 (2.5) | 2-10 | 5.5 (2.6) | 1-10 |
| **ADI-R** | Social Interaction | 10.6 (5.2) | 3-21 | 15.3 (5.6) | 4-24 | 14.1 (4.9) | 5-22 | 19.4 (5.9) | 8-27 |
|  | Communication | 10.1 (4.7) | 2-21 | 13.21 (3.9) | 5-20 | 10.3 (4.6) | 3-20 | 14.9 (3.8) | 8-23 |
|  | Restricted and Repetitive behavior | 3.1 (2.2) | 0-10 | 2.9 (1.7) | 0-7 | 3.25 (2.4) | 0-9 | 3.8 (2.3) | 0-7 |
|  | Total | 25.5 (10.3) | 9-55 | 33.1 (9.8) | 11-51 | 29.5 (10.6) | 8-46 | 40.1 (8.9) | 20-55 |
| **RCADS-C** | Social Phobia | 6.9 (3.7) | 0-15 | 7.3 (4.2) | 0-15 | 8.0 (4.4) | 1-16 | 7.0 (3.7) | 1-15 |
|  | Total anxiety | 20.3 (10.3) | 2-42 | 21.8 (13.5) | 3-54 | 22.1 (11.2) | 6-44 | 22.8 (11.2) | 6-47 |
| **SNAP-IV-P** | ADHD Inattention | 10.3 (6.7) | 1-24 | 13.2 (6.3) | 5-26 | 13.3 (7.0) | 2-26 | 14.7 (5.6) | 6-24 |
|  | ADHD hyperactivity/impulsivity | 8.7 (5.1) | 1-20 | 9.9 (7.3) | 0-27 | 8.3 (5.1) | 0-18 | 10.9 (4.8) | 3-19 |
| **IQ** | Verbal | 107.4 (14.3) | 80-132 | 101.8 (16.7) | 73-139 | 101.8 (18.1) | 74-145 | 98.4 (14.6) | 72-121 |
|  | Performal | 99.5 (19.8) | 70-133 | 96.8 (15.3) | 72-139 | 97.7 (17.1) | 60-126 | 98.2 (14.6) | 76-135 |
|  | Total | 104 (16.2) | 73-135 | 99.4 (14.7) | 77-132 | 99.5 (16.9) | 72-123 | 98.1 (15.1) | 77-128 |
